# Benzene induces haematotoxicity by promoting deacetylation and autophagy

**DOI:** 10.1111/jcmm.14003

**Published:** 2018-11-08

**Authors:** Shanhu Qian, Yixiang Han, Yifen Shi, Wanling Xu, Yiyi Zhu, Songfu Jiang, Yi Chen, Zhijie Yu, Si Zhang, Yiping Yang, Kang Yu, Shenghui Zhang

**Affiliations:** ^1^ Department of Hematology Wenzhou Key Laboratory of Hematology The First Affiliated Hospital of Wenzhou Medical University Wenzhou China; ^2^ Central Laboratory The First Affiliated Hospital of Wenzhou Medical University Wenzhou China; ^3^ Key Laboratory of Glycoconjugate Research Ministry of Public Health Department of Biochemistry and Molecule Biology Shanghai Medical College Fudan University Shanghai China; ^4^ Department of Medicine Duke University Medical Center Durham NC USA

**Keywords:** acetylation, autophagy, benzene, haematotoxicity, p300

## Abstract

Chronic exposure to benzene is known to be associated with haematotoxicity and the development of aplastic anaemia and leukaemia. However, the mechanism underlying benzene‐induced haematotoxicity, especially at low concentrations of chronic benzene exposure has not been well‐elucidated. Here, we found that increased autophagy and decreased acetylation occurred in bone marrow mononuclear cells (BMMNCs) isolated from patients with chronic benzene exposure. We further showed in vitro that benzene metabolite, hydroquinone (HQ) could directly induce autophagy without apoptosis in BMMNCs and CD34^+^ cells. This was mediated by reduction in acetylation of autophagy components through inhibiting the activity of acetyltransferase, p300. Furthermore, elevation of p300 expression by Momordica Antiviral Protein 30 Kd (MAP30) or chloroquine reduced HQ‐induced autophagy. We further demonstrated that in vivo, MAP30 and chloroquine reversed benzene‐induced autophagy and haematotoxicity in a mouse model. Taken together, these findings highlight increased autophagy as a novel mechanism for benzene‐induced haematotoxicity and provide potential strategies to reverse this process for therapeutic benefits.

## 1 INTRODUCTION

Chronic exposure to either high‐ or low‐dose benzene, a widely used industrial chemical and omnipresent environmental pollutant, can cause bone marrow suppression and may result in various diseases including aplastic anaemia, myelodysplastic syndrome, and leukaemia.^1^ It has been suggested that benzene metabolites can cause damage to haematopoietic cells via multiple mechanisms such as chromosomal aberration and covalent binding, epigenetic regulation, oxidative stress, and disruption of tumour surveillance.^2^ We have also shown that exposure to high‐dose benzene or its metabolite hydroquinone (HQ) leads to haematopoietic toxicity through induction of apoptosis in vivo and in vitro.^3^, ^4^ However, it remains unknown how low concentration of benzene exposure also results in haematopoietic toxicity.

Autophagy, first characterized in lower organisms, is an evolutionary conserved process. A portion of the cytosol is sequestered by a so‐called isolation membrane, formation of a double‐membrane structure called the autophagosome, which subsequently fuses with the lysosome/vacuole.^5^ Although physiological levels of autophagy are essential for the maintenance of cellular homeostasis during various stress conditions,^6^, ^7^, ^8^ excessive or uncontrolled levels of autophagy are able to induce cell death.^9^, ^10^, ^11^, ^12^, ^13^ Therefore, autophagy is a double‐edged sword, which has a protective role against cell death or contributes to autophagic cell death.

Autophagy can be regulated by posttranslational modifications including phosphorylation, ubiquitination, and acetylation.^12^, ^14^, ^15^ Our previous work has shown that hypoacetylation of histone H3 and H4 in Topo IIα promoter can lead to reduced expression of Topo IIα in patients with chronic benzene exposure,^16^ but the relationship of between hypoacetylation and autophagy remains unknown. Accumulating evidence shows that acetylation of autophagic components is rather complex and mainly regulated by a major acetyltransferase p300 and a NAD^+^‐dependent deacetylase Sirt1.^14^, ^17^, ^18^ p300 has been reported to be required for autophagy and capable of acetylating ATG7, LC3, and ATG3.^19^, ^20^, ^21^ The acetylation of histone H3 can reflect the activity of p300 as p300 directly acetylates histone H3 on lysine 56.^22^ The role of LC3 acetylation in autophagy has been well‐identified previously.^14^, ^17^, ^23^ Upon autophagy induction, the ATG4‐processed form of LC3, referred to as LC3‐I, is conjugated to phosphatidylethanolamine (PE) to form LC3‐II that attracts the autophagic substrate p62/SQSTM to the autophagosome, and lastly, p62/SQSTM protein sustains degradation during autophagosome maturation.^24^

In this study, we first demonstrate that increased autophagy and deacetylation of autophagic protein LC3 occur in bone marrow mononuclear cells (BMMNCs) isolated from patients with chronic low‐dose benzene exposure. Treatment of BMMNCs with low concentrations of benzene metabolite, HQ leads to deacetylation of autophagy components and subsequent autophagy via inhibiting p300. We further show that Momordica Antiviral Protein 30 Kd (MAP30), a ribosome‐inactivating protein isolated from dietary bitter melon, can increase p300 and reverse the autophagy induced by HQ. Finally, chronic exposure to low‐dose benzene in mice results in deacetylation, increased autophagy and haematopoietic toxicity, which can be reversed by treatment with MAP30 and a classical autophagy inhibitor chloroquine (CQ).

## 2 MATERIALS AND METHODS

### 2.1 Patients and healthy donors

All seven patients, three females and four males, were shoe workers and had experienced low exposure to benzene for at least 9 months, diagnosed as occupational benzene poisoning (National Standard GBZ68‐2002, China). The patient information was shown in Table S1. Twenty age‐matched healthy donors were enrolled as controls. This study was carried out with approval from the Ethics Committee of the First Affiliated Hospital of Wenzhou Medical University and written consents were obtained from all subjects participated in this study in accordance with the Declaration of Helsinki protocol.

### 2.2 Cell separation and HQ exposure

Mononuclear cells were isolated from bone marrow aspirations from healthy donors or patients with chronic benzene exposure using Ficoll‐hypaque and cultured in RPMI 1640 medium with 20% foetal bovine serum (both from Gibco, Grand Island, NY, USA). Also, BMMNCs had not been used for the following experiments until the percentage of CD38^+^ cells was more than 70% using flow cytometric analyses of the expression of CD34 and CD38. The CD34^+^ cells were isolated using a CD34 Microbead Kit (Miltenyi Biotec, Bergisch Gladbach, Germany) and cultured in RPMI 1640 medium with 20% foetal bovine serum to a density of 1 × 10^6^ cells/ml. The cells were treated with HQ (Jinshan chemical reagent factory, Zhejiang, China) at final concentrations of 5, 10, and 20 μmol/L for 12 hours. And the cells were also treated with C646 or Acetyl‐CoA (both from Sigma‐Aldrich, St.Louis, MO, USA).

### 2.3 Cell viability assay

Trypan blue is cell membrane impermeable and only enters cells with damaged membrane. Therefore, the cell viability can be determined using trypan blue exclusion assay (Beyotime Institute of Biotechnology, Haimen, Jiangsu, China). Briefly, the cells were stained with trypan blue solution and counted using an inverted microscope (Olympus, Tokyo, Japan) within 5 minutes. The dead cells were stained with blue colour and the cell viability was calculated by the following formula: cell viability (%) = (1 − the dead cell number/the total number) × 100%.

### 2.4 Apoptosis assay

The annexin V and propidium iodide (PI) double staining method was used to investigate whether low concentration of HQ induces apoptosis of BMMNCs. Briefly, after treatment with HQ for 12 hours, the cells were collected and incubated with FITC‐labelled annexin V and PI for 15 minutes and then analysed with CellQuest software on a flow cytometry (FACSCalibur; BD, Mountain View, CA, USA).

### 2.5 Immunoprecipitation and Immunoblotting

The cells were collected and added immunoprecipitation buffer (Beyotime Institute of Biotechnology) to prepare protein lysates. The protein lysates were mixed with the indicated primary antibody overnight for 4°C, followed by incubation with 20 μL of protein G + A‐Sepharose (Bioworld Technology, St. Louis Park, MN, USA) for 2 hours at 4°C. The immunoprecipitates were collected and washed three times with PBS and subsequently boiled in 40 μL 1× sample buffer for 5 minutes and analysed using SDS‐PAGE. After proteins were transferred onto a PVDF membrane (Millipore, Bedford, MA, USA), the membrane was blocked with 5% nonfat dried milk in tris‐buffered saline with tween^®^ 20 (TBST) for 1 hour, followed by detection of the indicated primary antibodies (LC‐3, p62, ATG7, ATG3, Beclin 1, and β‐actin were bought from cell signaling technology, Beverly, MA, USA, 1:1000; H3, and ac‐H3 were bought from Millipore, 1:1000; TBP was bought from Bioworld Technology, 1:1000; p300 was bought from Santa Cruz Biotechnology, Santa Cruz, CA, USA, 1:250). After washing, the membrane was incubated with appropriate horseradish peroxidase‐conjugated secondary antibodies (Cell Signaling Technology) at 37°C for 1 hour. Bands were visualized by enhanced chemiluminescence reagent (Thermo fisher, Fremont, CA, USA).

### 2.6 Immunofluorescence

The cells were collected and fixed by 4% paraformaldehyde for 10 minutes and permeabilized with 0.05% Triton X‐100 for 30 minutes and subsequently blocked with 5% bovine serum albumin in PBS for 30 minutes and immunostained with anti‐LC3 or anti‐acetylated lysine (both from Cell Signaling Technology) overnight at 4°C. After washing, the cells were incubated with FITC‐labelled goat anti‐rabbit IgG for 1 hour and DAPI or PI for 15 minutes, respectively. Finally, images were recorded using a fluorescence microscope (BX51; Olympus, Tokyo, Japan).

### 2.7 Quantitative real‐time RT‐PCR for gene expression analysis

Quantitative real‐time RT‐PCR was used to analyse the expression of p300 and a reference gene GAPDH as described previously.^25^ The sequences of specific primers see Table S2.

### 2.8 RNA interference targeting ATG5 and p300

The cells were transfected with ATG5 siRNA, Negative control FAM (NC FAM), p300 siRNA, or nontargeting control siRNA (UNisiRNA) (Sangon Biotech, Shanghai, China) using lipofectamine^®^ 3000 (Invitrogen, Eugene, OR, USA) according to the manufacturer's instructions. After 48 hours, the cells were collected and analysed. The sequences of specific siRNAs see Table S3.

### 2.9 Benzene‐induced haematotoxicity mouse model

The mouse model of benzene‐induced haematotoxicity was constructed according to our previous described method with minor modifications.^3^ Briefly, ninety male ICR mice weighting 23‐25 g were obtained from Laboratory Animal Centre of Wenzhou Medical University and randomized into corn oil group (2 mL/kg), benzene group (benzene 1 mL/kg; corn oil 1 mL/kg), benzene + MAP30 group (benzene 1 mL/kg; corn oil 1 mL/kg; MAP30 0.5 mg/kg), MAP30 group (corn oil 2 mL/kg; MAP30 0.5 mg/kg), benzene + CQ group (benzene 1 mL/kg; corn oil 1 mL/kg; CQ 50 mg/kg), CQ group (corn oil 2 mL/kg; CQ 50 mg/kg), 15 mice in each group. MAP30 was kindly provided by professor Meng from Chengdu Medical College.^26^ These mice were subcutaneously injected with the mixture of benzene (Jinshan chemical reagent factory) and corn oil (COFCO, Beijing, China), and meanwhile intraperitoneally injected with MAP30 or CQ (Sigma‐Aldrich) three times a week for a total of 12 times. Then, these mice were killed under anaesthesia, and the femurs and tibias were removed quickly. Bone marrow was flushed out with RPMI 1640 media by use of a 26‐gauge needle and BMMNCs were isolated using Ficoll density gradient centrifugation.

### 2.10 Blood cell counts

Blood cell counts were assessed as described previously.^3^, ^27^ Briefly, the level of haemoglobin was determined using a haemoglobin analyser (Mission, Sichuan, China). White blood cells (WBC) were quantified under a light microscopy after blood was diluted in Red Blood Cell Lysis Buffer (Solarbio, Beijing, China). Platelets were quantified under a phase‐contrast microscope after blood was diluted with 1% ammonium oxalate.

### 2.11 Statistical analysis

The data were presented as mean ± SEM and analysed by *t* test or one‐way ANOVA followed by a post hoc Turkey's test to determine the differences between the groups. Differences were considered significant at *P* < 0.05.

## 3 RESULTS

### 3.1 Increased autophagy and reduced acetylation in haematopoietic cells of patients with chronic benzene exposure

**Figure 1:**
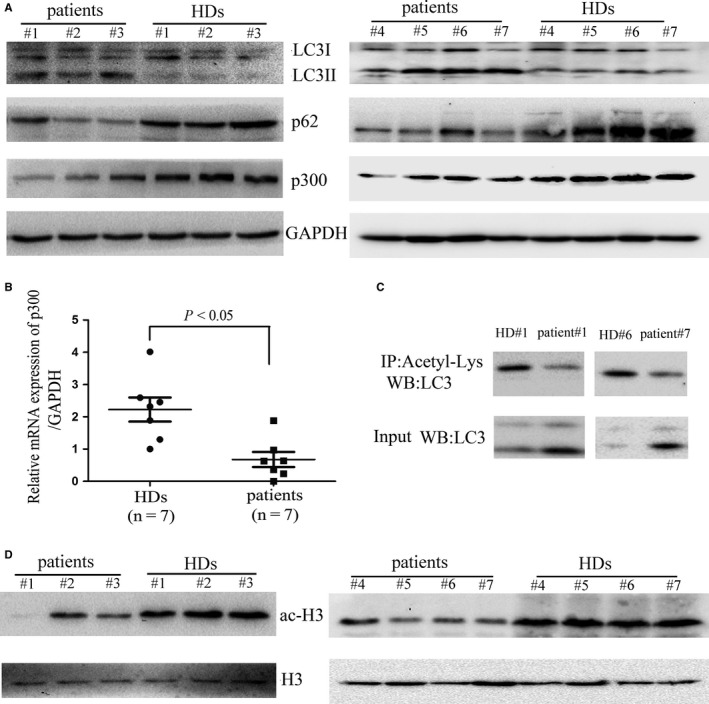
Decreased acetylation of LC3 and increased autophagy are shown in BMMNCs from patients with chronic benzene exposure. (A) Bone marrow samples were extracted from healthy donors (HDs) and patients with chronic benzene exposure, and mononuclear cells were subsequently separated using Ficoll‐hypaque and solubilized and immunoblotted with antibodies against p300, LC3, p62, or GAPDH, respectively. (B) Total RNA was isolated from BMMNCs isolated from patients with chronic benzene exposure or HDs, and reverse transcribed into cDNA and subsequently quantitative real‐time RT‐PCR for p300 and GAPDH. (C) The acetylation level of endogenous LC3 was determined by immunoprecipitation with the antibody of acetyl‐lysine, followed by western blot analysis of LC3. (D) The acetylation level of histone H3 was determined using western blot. Images shown are representatives of seven patients with chronic benzene exposure and seven HDs

We first observed that all seven enrolled patients with exposure to benzene for at least 9 months had obvious symptoms of haematotoxicity (Table S1). To address the role of autophagy pathway in the development of haematotoxicity, we analysed whether benzene exposure influences the level of autophagy components in BMMNCs. In this study, BMMNCs mainly consisted of haematopoietic progenitor cells (Figure S1A). We observed that the level of the autophagy marker, LC3‐II increased, whereas that of p62, the substrate for LC3, decreased in BMMNCs from patients with chronic benzene exposure compared with those from healthy donors (Figure 1A, Figure S1B and C), suggesting that an increased autophagic flux does occur in patients with chronic benzene exposure. The increase in autophagic flux was associated with a decrease in both the protein (Figure 1A and Figure S1D) and mRNA (Figure 1B) levels of p300 in BMMNCs from patients with chronic benzene exposure. Furthermore, we found that acetylation of LC3 was predominant in LC3‐I rather than LC3‐II (Figure 1C), and the acetylation of LC3 (Figure 1C) and histone H3 (Figure 1D) were decreased in BMMNCs from patients with chronic benzene exposure compared with those from healthy donors. Our results suggested that there is a decreased acetyltransferase activity of p300 in BMMNCs from patients with chronic benzene exposure. Taken together, our clinical data suggested that chronic low‐dose benzene exposure is associated with an increase in autophagic flux as a result of a decrease in acetyltransferase p300 and acetylation of autophagic marker protein LC3 in haematopoietic cells.

### 3.2 Benzene metabolite, HQ induces autophagy without apoptosis in BMMNCs and CD34^+^ cells

**Figure 2:**
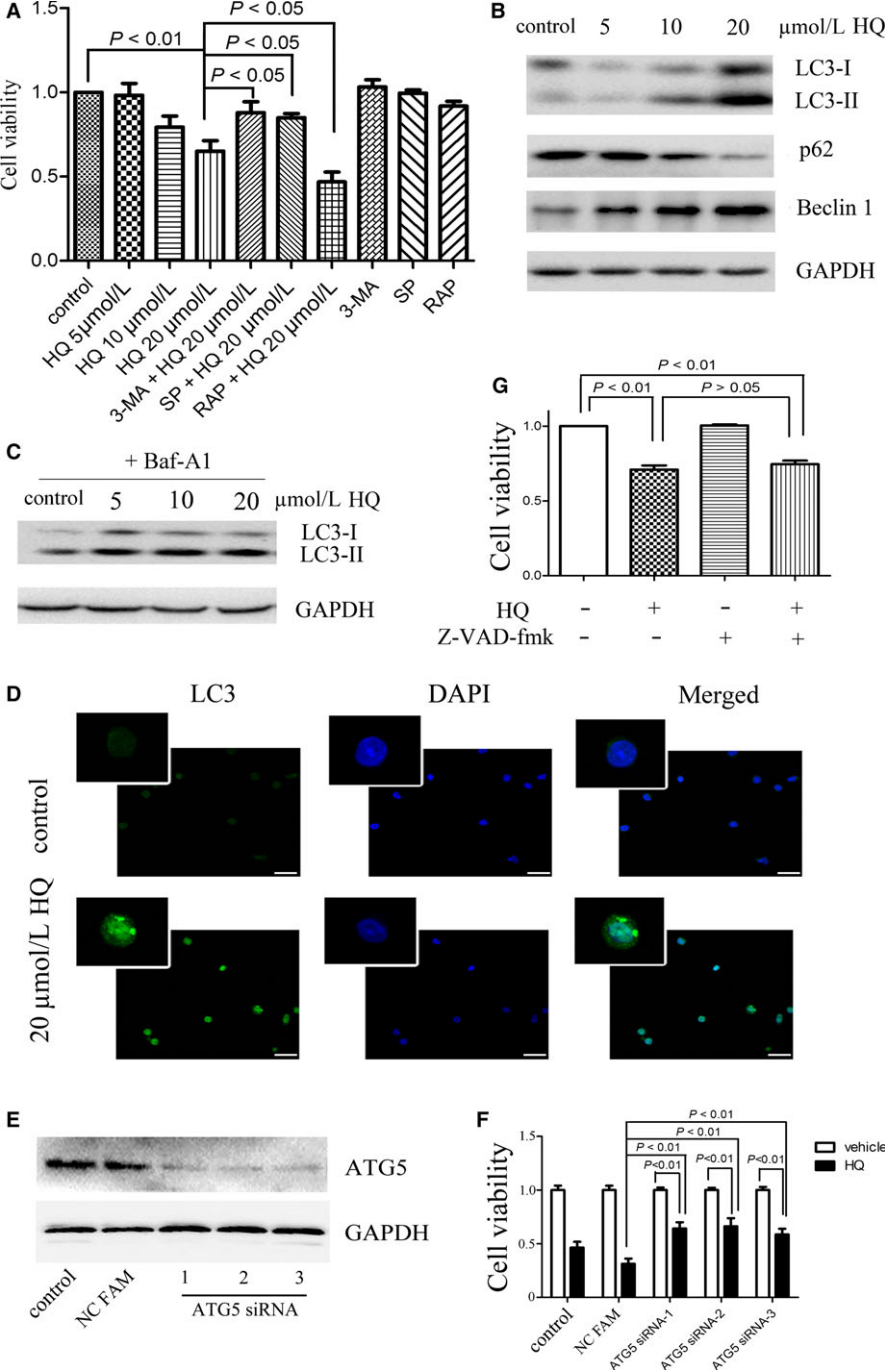
HQ increases autophagic flux without apoptosis. (A) BMMNCs were pretreated with or without classical autophagic activator rapamycin (RAP, 50 nmol/L) or autophagic inhibitor 3‐MA (5 mmol/L) or Spautin‐1 (SP, 10 μmol/L) for 30 min, and treated with various concentrations of HQ for 12 h. Then, the cell viability was determined using trypan blue exclusion assay. (B) The cells were treated with various concentrations of HQ for 12 h and solubilized and immunoblotted with antibodies against LC3, p62, Beclin 1, and GAPDH, respectively. (C) BMMNCs were treated with various concentrations of HQ for 12 h in the presence of lysosomal inhibitor bafilomycin A1 (Baf‐A1, 20 nmol/L) and solubilized and immunoblotted with antibodies against LC3 and GAPDH, respectively. (D) Treatment with 20 μmol/L HQ for 12 h enhanced LC3 expression by immunofluorescence with the antibody of LC3 and DAPI; scale bar = 30 μm. (E, F) ATG5 was silenced using three specific siRNAs, and silencing of ATG5 significantly attenuated HQ‐induced cell viability inhibition. (G) Pretreatment with Z‐VAD‐fmk, a pan‐caspase inhibitor unaffected HQ‐induced cell viability inhibition. Statistical data and image representing four independent experiments from nine different donors were shown

The observation that patients with chronic benzene exposure had increased autophagic flux in BMMNCs prompted us to investigate whether treatment of benzene metabolite HQ directly promotes autophagy in vitro. We showed that treatment with low concentrations of HQ for 12 hours inhibited the viability of BMMNCs (Figure 2A) and CD34^+^ haematopoietic progenitor cells (Figure S2A) dose‐dependently. We further showed that inhibition of autophagy by 3‐methyladenine (3‐MA) or Spautin‐1 (SP) attenuated the effect of HQ, whereas activation of autophagy by rapamycin further enhanced the effect of HQ (Figure 2A). We also found that HQ treatment promoted the expression of autophagy‐associated markers including LC3‐II and Beclin 1 at a dose‐dependent manner in BMMNCs (Figure 2B) and CD34^+^ cells (Figure S2B). The expression of p62 was decreased after treatment with HQ, further supporting the notion that HQ induced autophagic flux. Furthermore, the expression of LC3‐II was also significantly increased in the presence of bafilomycin A1 (Baf‐A1) (Figure 2C), a specific inhibitor of the vacuolar ATPase required for the fusion between autophagosomes and lysosomes. And we also found that HQ treatment increased LC3 expression in BMMNCs using immunofluorescence (Figure 2D and Figure S3A). Additionally, HQ treatment promoted AMPK activation and inhibition of mTOR signalling (Figure S3B), a classical signalling pathway implicated in autophagy regulation, further confirmed that HQ did induce autophagy in BMMNCs. To further confirm the role of autophagy pathway in HQ‐induced cytotoxicity, we examined the effect of silencing of ATG5, an autophagy‐related gene. We showed that siRNA silencing of ATG5 (Figure 2E) significantly inhibited the cytotoxicity of HQ on BMMNCs (Figure 2F).

To address whether apoptosis may play a role, we treated BMMNCs with low concentrations of HQ for 12 hours and found that the treatment did not promote the apoptosis of BMMNCs (Figure S3C) by Annexin V and PI staining. Furthermore, pretreatment with Z‐VAD‐fmk, a pan‐caspase inhibitor involved in apoptosis did not affect the HQ‐induced suppression of cell viability in BMMNCs (Figure 2G). Taken together, these data suggest that low concentrations of HQ directly promotes autophagy without apoptosis in BMMNCs.

### 3.3 HQ reduces acetylation of autophagy components in BMMNCs

**Figure 3:**
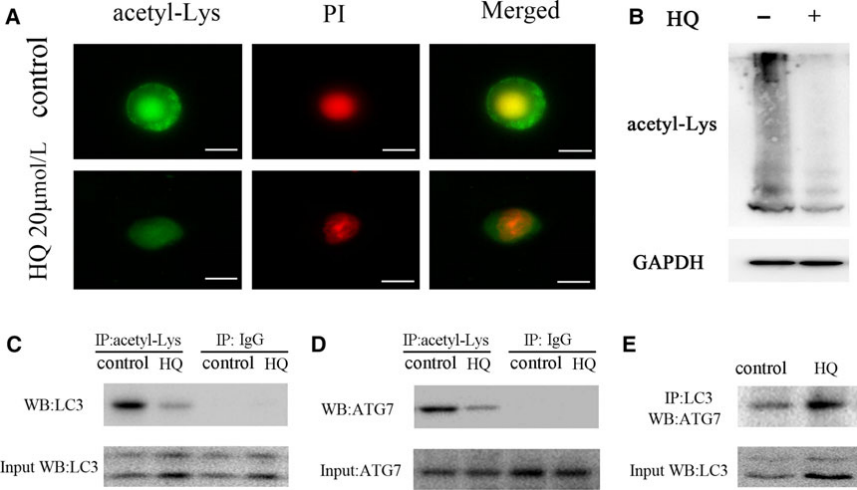
HQ induces deacetylation of cellular proteins. (A) Treatment with HQ 20 μmol/L for 12 h suppressed the total acetylation levels in BMMNCs using immunofluorescence with anti‐acetyl lysine antibody; scale bar = 10 μm. (B) Western blot showed that HQ treatment reduced the total acetylation levels in BMMNCs. (C, D) HQ reduced the acetylation levels of LC3 and ATG7 in BMMNCs. The acetylation of autophagy components was assessed by immunoprecipitation (IP) with the antibody against acetyl‐lysine, followed by western blot of ATG7 and LC3. (E) The interaction of LC3 and ATG7 was determined by IP with the antibody against LC3, followed by western blot of ATG7. Representative images were shown from three independent experiments from five different donors

Patients with chronic benzene exposure showed decreased acetylation in BMMNCs (Figure 1), which prompts us to investigate whether benzene metabolite HQ directly inhibits acetylation. Indeed, HQ treatment significantly inhibited the overall lysine acetylation of cellular proteins of BMMNCs (Figure 3A and B, and Figure S3D). Obviously, acetylation of LC3 was predominant in LC3‐I, and HQ treatment significantly reduced the acetylation of LC3 (Figure 3C). Another important autophagy components, ATG7, its acetylation was also markedly attenuated after HQ treatment (Figure 3D), suggesting that deacetylation of autophagy components may contribute to autophagy. Furthermore, the deacetylation of LC3 and ATG7 promoted the physical interaction between ATG7 and LC3 (Figure 3E), which could further promote autophagy. Taken together, these data suggested that HQ treatment could decrease the overall lysine acetylation of cellular proteins, especially autophagic proteins ATG7 and LC3 in BMMNCs.

### 3.4 HQ promotes autophagy by inhibiting the activity of p300

**Figure 4:**
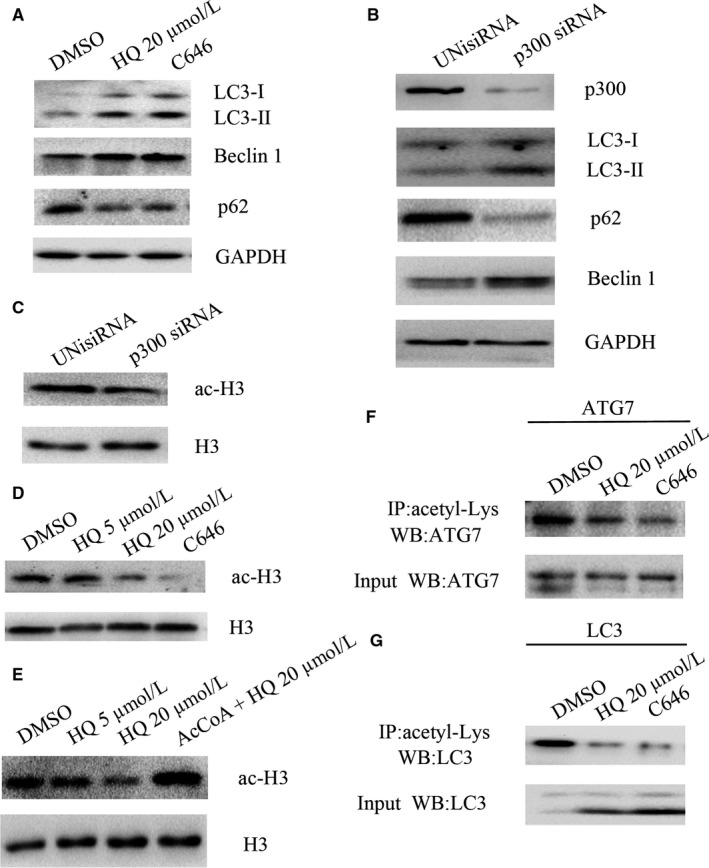
HQ induces autophagy by inhibiting the activity of p300. (A) BMMNCs were treated with 20 μmol/L HQ or an antagonist of p300 C646 (10 μmol/L) for 12 h and subsequently collected and immunoblotted with antibodies against LC3, p62, Beclin 1, or GAPDH. (B) BMMNCs were transfected with p300 siRNA or control UNisiRNA using lipofectamine^®^ 3000. After 48 h, the cells were collected and solubilized and immunoblotted with antibodies against LC3, p62, Beclin 1, p300, or GAPDH. (C) p300 silencing using siRNA inhibited acetylation of histone H3, one of p300 preferred substrates. (D) Treatment with HQ 20 μmol/L or C646 for 12 h significantly inhibited acetylation of histone H3. (E) Acetyl‐CoA (AcCoA) 100 μmol/L significantly reverted the inhibition of acetylation of histone H3 induced by HQ 20 μmol/L in BMMNCs. (F, G) BMMNCs were treated with HQ 20 μmol/L or C646 for 12 h and subsequently collected and solubilized and immunoprecipitated with the antibody against acetyl‐lysine, followed by western blot of ATG7 and LC3. Representative images were shown from four independent experiments from seven different donors

The reduced acetylation of autophagy components by HQ treatment prompted us to next investigate whether autophagy is regulated by acetyltransferase p300. We showed that C646,^28^ a “specific” antagonist of p300, significantly increased LC3‐II and Beclin 1, accompanied by a decrease in p62 expression at a concentration of 10 μmol/L, even more significant than HQ at a concentration of 20 μmol/L (Figure 4A), suggesting that C646 induces autophagy by p300 inhibition in BMMNCs. This was further verified by knockdown of p300 using siRNA, which induced similar levels of autophagy to that of C646 (Figure 4B).

Both C646 and p300 siRNA decreased the acetylation of histone H3 in BMMNCs (Figure 4C and D). HQ treatment also decreased the acetylation of histone H3 in a dose‐dependent manner. However, the effect was completely inhibited by addition of acetyl‐CoA at a concentration of 100 μmol/L (Figure 4E). C646 as an inhibitor of p300 significantly decreased the acetylation of LC3 and ATG7, which was more obvious than HQ did (Figure 4F and G). Taken together, these data verified that HQ promoted autophagy by inhibiting the activity of p300.

### 3.5 Elevation of p300 expression by MAP30 reduces HQ‐induced autophagy

**Figure 5:**
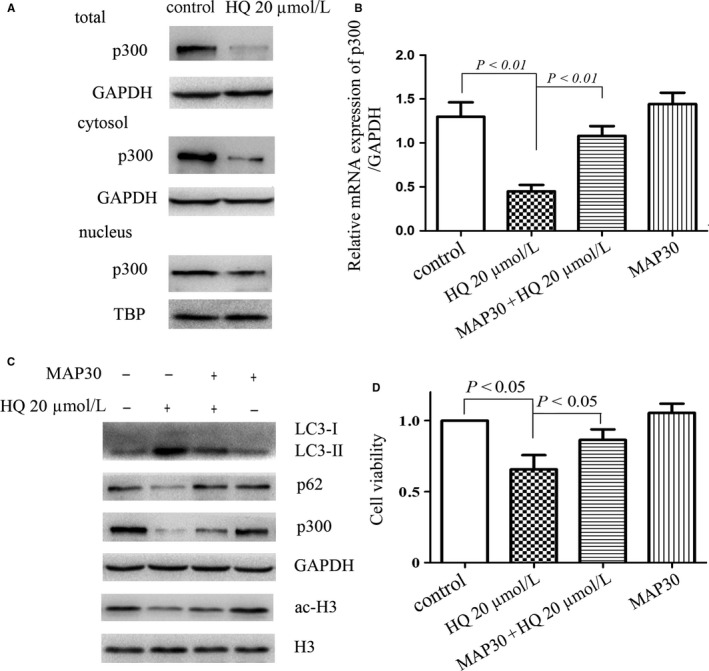
MAP30 restores decreased expression of p300 induced by HQ. (A) Treatment with HQ 20 μmol/L for 12 h significantly decreased the level of p300 in both the cytosol and nucleus in BMMNCs. TBP (TATA‐binding protein) as a nuclear loading control was used. (B) BMMNCs were treated with HQ 20 μmol/L in the presence or absence of MAP30 (25 nmol/L) for 12 h. Total RNA was subsequently extracted and reverse transcribed into cDNA, and real‐time PCR was performed to obtain the relative levels of mRNA for p300. (C) MAP30 inhibited the autophagy flux induced by HQ 20 μmol/L via reverting decreased p300 and acetylation of histone H3. (D) The cell viability was determined using trypan blue exclusion assay in BMMNCs treated with or without HQ (20 μmol/L) and MAP30 (25 nmol/L) for 12 h. Statistical data and images representing three independent experiments from five different donors were shown

We next investigated whether HQ affects the expression or distribution of p300. As shown in Figure 5A, HQ significantly decreased the expression of p300 in both cytosol and nucleus fraction of BMMNCs, supporting the inhibition of acetyltransferase activity because of the reduction in p300 expression mostly. Real‐time RT‐PCR data unearthed that HQ inhibited p300 expression at the transcriptional level (Figure 5B). Additionally, we investigated whether HQ treatment influences other acetyltransferase's expression, and found that HQ almost did not influence the expression of Sirt1 (Figure S3E), a main deacetylatransferase, in BMMNCs.

We further investigated whether modulation of p300 expression regulates the autophagic flux induced by HQ. Our previous study have found that MAP30 enhances p300 expression in acute myeloid leukaemia cells.^29^ As expected, MAP30 increased p300 expression and attenuated autophagy and cell death induced by HQ in BMMNCs (Figure 5C and D).

### 3.6 MAP30 or CQ reverses benzene‐induced autophagy and haematotoxicity in vivo in a mouse model

**Figure 6:**
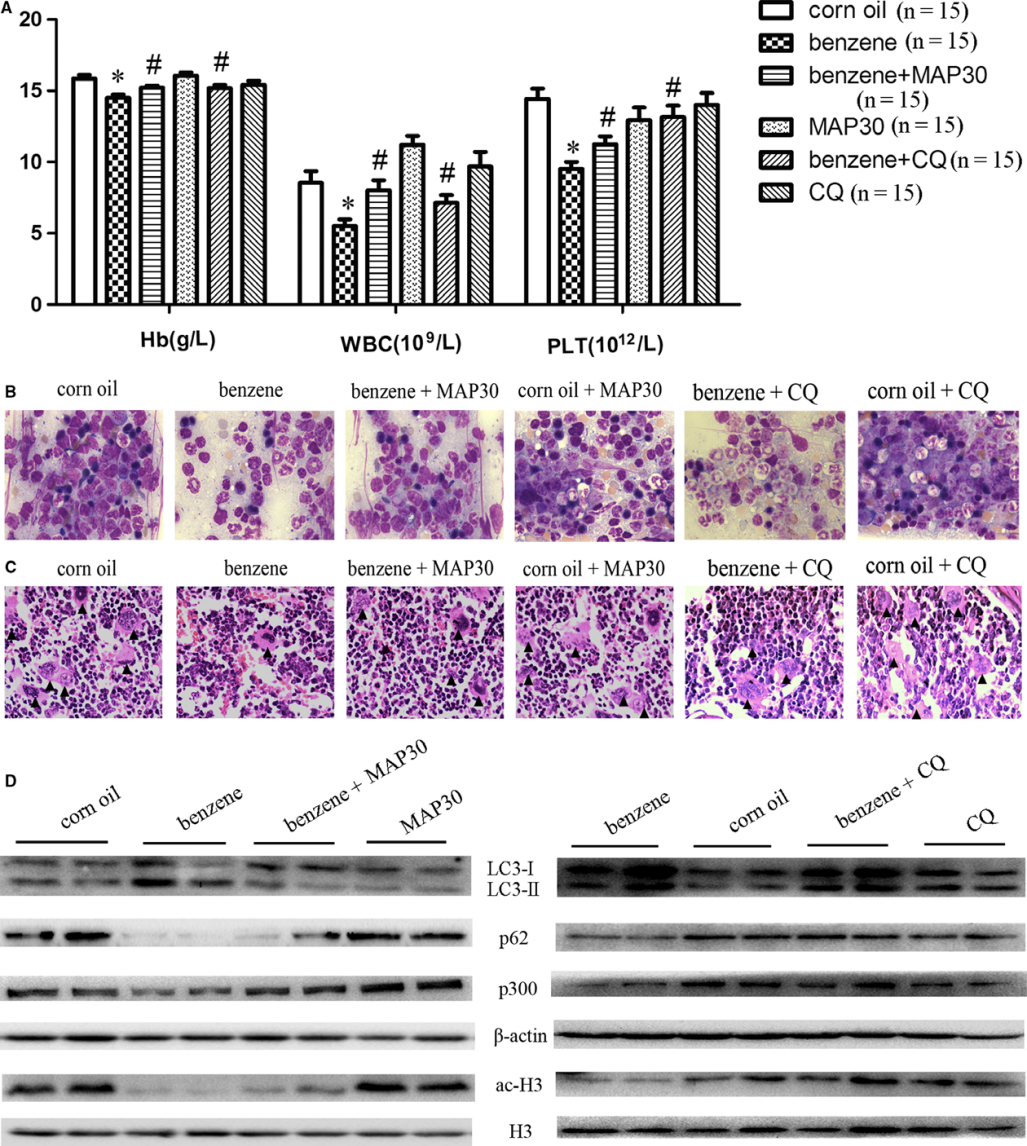
Benzene induces autophagy and deacetylation in BMMNCs in a mouse model, and MAP30 or chloroquine (CQ) relieves benzene‐induced haematotoxicity. ICR mice weighting 23‐25 g were randomized into benzene group (benzene 1 mL/kg; corn oil 1 mL/kg), corn oil group (2 mL/kg), benzene + MAP30 group (benzene 1 mL/kg; corn oil 1 mL/kg; MAP30 0.5 mg/kg), and MAP30 group (corn oil 2 mL/kg; MAP30 0.5 mg/kg), benzene + CQ group (benzene 1 mL/kg; corn oil 1 mL/kg; CQ 50 mg/kg), and CQ group (corn oil 2 mL/kg; CQ 50 mg/kg), 15 mice in each group. After subcutaneous injection with the mixture of benzene and corn oil and intraperitoneal injection with MAP30 or CQ three times a week for a total of 12 times, blood was obtained from each mouse and the mice were subsequently killed under anaesthesia. (A) The level of haemoglobin (Hb), and white blood cells (WBC) and platelet (PLT) counts were evaluated in each groups. (B) Bone marrow aspirates were flushed out, and then, the smears were observed using Wright staining. Reduced granulocytes and erythrocytes were seen in benzene‐treated mice. (C) The femurs were separated and embedded in paraffin and subsequently cut into slices with a thickness of 5 μm, which was observed using HE staining, “▲” means megakaryocyte. (D) Bone marrow was flushed out using RPMI 1640 and mononuclear cells were separated using Ficoll‐hypaque. Then, the autophagy‐related markers, p300 and the acetylation of histone H3 were assessed using western blot analyses. The different mice in benzene group and corn oil group were used as representatives of the expression of indicated proteins in the left and right panel. Statistical data and images representing five independent experiments from 15 mice each group were shown

To study whether MAP30 can protect benzene‐induced haematotoxicity in vivo, we established a mouse model of low‐dose benzene‐induced haematotoxicity. We found that the level of haemoglobin, and the numbers of WBC and platelets decreased significantly in benzene‐treated mice compared with the control mice, and these three indicators were at least partially reversed in the group of benzene + MAP30 and benzene + CQ compared with those in benzene‐treated mice (Figure 6A), suggesting a potential therapeutic role of MAP30 in benzene‐induced haematotoxicity. Haematotoxicity was also verified by the analysis of bone marrow aspiration, which demonstrated that granulocytes and erythrocytes were decreased and lymphocytes were increased in benzene‐treated mice, and treatment with CQ or MAP30 attenuated these effects on haematopoietic cells induced by benzene (Figure 6B). The pathological examination of the femurs also demonstrated that megakaryocytes were reduced in the bone marrow of benzene‐treated mice, and CQ or MAP30 partially recovered the counts of megakaryocytes in benzene‐treated mice (Figure 6C and Figure S4A).

We next determined whether autophagy is the major mechanism of low dose of benzene‐induced haematotoxicity. As shown in Figure 6D, LC3‐II significantly increased and p62 markedly decreased in BMMNCs from benzene‐treated mice, and the benzene + MAP30 group or the benzene + CQ group reduced the level of autophagy, suggesting that benzene leads to haematotoxicity via autophagy induction and can be reversed by MAP30 or CQ treatment. Furthermore, the levels of p300 mRNA and protein were also significantly decreased in benzene‐treated mice (Figure S4B and Figure 6D), which led to a robust decrease in the acetylation of histone H3, and MAP30 or CQ could attenuate it (Figure 6D). Additionally, the acetylation of ATG7 was also decreased in benzene‐treated mice compared with the control mice (Figure S4C). No significant apoptosis was observed in BMMNCs isolated from benzene‐treated mice compared with those from control mice (Figure S4D), suggesting that induction of apoptosis is not the major mechanism of low dose of benzene‐induced haematotoxicity. Taken together, these data suggested that benzene causes haematotoxicity via p300 inhibition‐mediated autophagy induction, which can be reversed by both MAP30 and CQ treatment.

## 4 DISCUSSION

Increasing evidence demonstrates that deregulated autophagy may critically influence vital cellular processes such as programmed cell death and thereby may play a key role in the pathogenesis of many diseases in humans. However, the underlying mechanism remains unknown. To our knowledge, in the present study, we have demonstrated for the first time that increased autophagy occurs in BMMNCs from both patients with chronic benzene exposure and benzene‐treated mice, with decreased expression and activity of p300 at the transcriptional level. Further insight into mechanism of action revealed that its metabolite HQ promoted autophagy via inhibiting p300.

Benzene is a widely distributed environmental contaminant. Although a number of measures have been taken to reduce benzene contaminant, benzene exposure remains one of the most important health problems to be dealt with. Exposure to benzene results primarily in haematotoxicity including pancytopaenia, aplastic anaemia, thrombocytopaenia, and leukaemia, but the underlying mechanisms remain unclear. In the present study, increased autophagy and decreased p300 were observed in BMMNCs from seven patients with chronic benzene exposure, suggesting that autophagy induction and deacetylation may be the underlying mechanism by which benzene causes haematotoxicity.

Autophagy is a lysosomal pathway involved in degradation of long‐lived proteins and damaged organelles. It appears as an adaptation mechanism that is vital for cellular homeostasis in response to various stress conditions. However, excessive levels of cellular autophagy cause cell death, termed “autophagic cell death”, which means early degradation of organelles but preservation of cytoskeletal elements until late stages. In the present study, the autophagy inhibitor 3‐MA reversed the cell death caused by HQ and the autophagy activator rapamycin exaggerated the cell death cause by HQ, both verified that benzene metabolite HQ did induce autophagic cell death of BMMNCs in vitro, which differ from a previous study. Xu et al^30^ have showed that autophagy plays a cytoprotective role in HQ‐induced lymphoma TK cell death. Thus, we think that the role of autophagy in HQ‐induced cell death may be cell‐specific. Silencing of ATG5, but not Z‐VAD‐fmk, significantly inhibited HQ‐induced cell death, further confirming that low concentrations of HQ induce autophagic cell death but not apoptosis in BMMNCs. In benzene‐induced haematotoxicity mouse model, treatment with 1 mL/kg benzene for 4 weeks only promoted autophagy but not apoptosis, inconsistently with our previous study,^3^ in which treatment with 2 mL/kg benzene for 8 weeks promoted a marked apoptosis in BMMNCs.

Deregulation of autophagy is implicated in many human diseases, such as heart and liver diseases, cancers, myopathies, neurodegeneration, and gastrointestinal disorders. Mounting proteins and signalling pathways have been shown to play roles in the regulation of autophagy,^31^, ^32^ in which at present more attention is being paid to epigenetic modification, especially acetylation.^33^ Increasing evidence shows that acetylation of ATG genes can lead to autophagy activation or inhibition. For example, Sirt1 deacetylates LC3 in the nucleus and subsequently induces autophagy.^14^ The acetylation of Beclin 1 regulated by p300 and Sirt1 inhibits autophagosome maturation and promotes tumour growth.^18^ In the present study, patients with chronic benzene exposure demonstrated decreased acetylation of histone H3 in BMMNCs, consistent with our previous report.^16^ HQ reduced the acetylation of ATG7 and LC3 by inhibiting the activity of p300. Obviously, acetylation was predominantly in LC3‐I rather than LC3‐II in BMMNCs, and HQ treatment dramatically inhibited the acetylation of LC3 and subsequently enhanced the conjugation of LC3 to PE. Which type of LC3 acetylation implicating in the process of autophagy remains obscure so far, for example, serum starvation mainly led to deacetylation of LC3‐I in HEK293 cells,^14^ whereas in Hela cells, serum starvation profoundly decreased the acetylation of LC3‐II.^23^

The activity and expression of p300 were also inhibited by HQ or benzene both in the cytosol and nucleus. As p300 is a major endogenous repressor of autophagy, HQ acted as an autophagy inducer and reduced the acetylation of autophagy components including LC3 and ATG7 by inhibiting p300. HQ has been reported to dramatically enhance the Src kinase activity by directly targeting and binding to the sulphydryl group of cysteine‐483 (C483) and C400 residues of Src,^34^ which results in the disruption of intracellular disulphide bonds. How HQ regulate the expression of p300 remains largely unknown. Further works should be proceeded to clarify the mechanism how HQ decrease the expression of p300 at the transcriptional level in future studies. As we all know, activity of p300 depends on the concentration of acetyl‐CoA and meanwhile, acetyl‐CoA is also mostly regulated by p300.^35^, ^36^ So HQ deregulated this cycle and resulted in autophagy and eventually cell death.

MAP30 has been reported to possess strong antitumour activity against many tumour cells, so is α‐MMC.^26^, ^37^ Our previous study has shown that MAP30 at a high concentration 2 μmol/L inhibits autophagy through enhancing p300 and induces apoptosis in acute myeloid leukaemia cells.^29^ In this present study, we demonstrated that MAP30 at only 25 nmol/L could also attenuate HQ‐induced autophagy by increasing the expression of p300, further verified that the autophagy was regulated by p300. Benzene‐induced haematotoxicity were markedly reduced by intraperitoneal injection of MAP30. Furthermore, the autophagy inhibitor CQ as an anti‐malarial drug has been used extensively in clinical, and undoubtedly, it robustly relieved benzene‐induced haematotoxicity in vivo. Therefore, CQ and MAP30 may be two potential therapeutic drugs for patients with chronic benzene exposure.

To our knowledge, this is the first time to unveil the role of autophagy caused by benzene and the underlying mechanisms. HQ significantly inhibits p300 and subsequently induces excessive autophagy and eventually leads to cell death. Of note, the classical autophagy inhibitor CQ and MAP30 seem to hold great promise for treating with patients with chronic benzene exposure.

## CONFLICT OF INTEREST

The authors confirm that there are no conflicts of interest.

## AUTHOR CONTRIBUTION

YY, KY, and SHZ designed research; SQ, YH, YS, YZ, WX, and SHZ performed experiments; SQ, SJ, YC, ZY, SZ, YY, KY, and SHZ analysed the data; and SQ and SHZ wrote the paper.
