# Supplementary material for: Benzene induces haematotoxicity by promoting deacetylation and autophagy
Source: J Cell Mol Med. 2018 Nov 8;23(2):1022–33. doi: 10.1111/jcmm.14003 (PMC6349156; doi:10.1111/jcmm.14003)
Supplement: Supplementary file 1 [file JCMM-23-1022-s001.docx]

**Table S1.** The information of seven patients with chronic benzene exposure.

| patients | age | gender | [symptom](javascript:void(0);) | Exposure time |
| --- | --- | --- | --- | --- |
| Patient #1 | 23y | Male | pancytopenia | 1 year |
| Patient #2 | 35y | Male | thrombopenia | 10 years |
| Patient #3 | 31y | Female | pancytopenia | 4 years |
| Patient #4 | 22y | Female | anemia | 9 months |
| Patient #5 | 28y | Female | pancytopenia | 1 year |
| Patient #6 | 20y | Male | thrombopenia | 1.5 year |
| Patient #7 | 34y | Male | pancytopenia | 2 years |

**Table S2.** The sequences of the primers used for real-time qPCR.

|  | Forward primer | Reverse primer |
| --- | --- | --- |
| human p300 | 5’- AGGCTGTATCAGAGCGTAT -3’ | 5’- TGCTTTCATTGCTGGTGT -3’ |
| mouse p300 | 5’- GGATAATGCCCAATCAAGTCA-3’ | 5’- CCCTGCTGTAGTGGCTCAGT-3’ |
| human GAPDH | 5’- ATCATCAGCAATGCCTCC-3’ | 5’- CATCACGCCACAGTTTCC-3’ |
| mouse GAPDH | 5’- GGTTGTCTCCTGCGACTTCA-3’ | 5’-TGGTCCAGGGTTTCTTACTCC-3’ |

**Table S3.** The sequences of siRNAs.

|  | sequences (5’-3’) | |
| --- | --- | --- |
| p300 siRNA | CAGGUAUGAUGAACAGUCCAGUAAA | |
| UNisiRNA | UUCUCCGAACGUGUCACGUTT | |
| ATG5 siRNA-1 | GUGAUGAUUCAUGGAAUUGTT | CAAUUCCAUGAAUCAUCACTT |
| ATG5 siRNA-2 | GCAGUGGCUGAGUGAACAUTT | AUGUUCACUCAGCCACUGCTT |
| ATG5 siRNA-3 | GCUAGCUGGCUGUCCAUAUTT | AUAUGGACAGCCAGCUAGCTT |
| Negative control | UUCUCCGAACGUGUCACGUTT | ACGUGACACGUUCGGAGAATT |

**
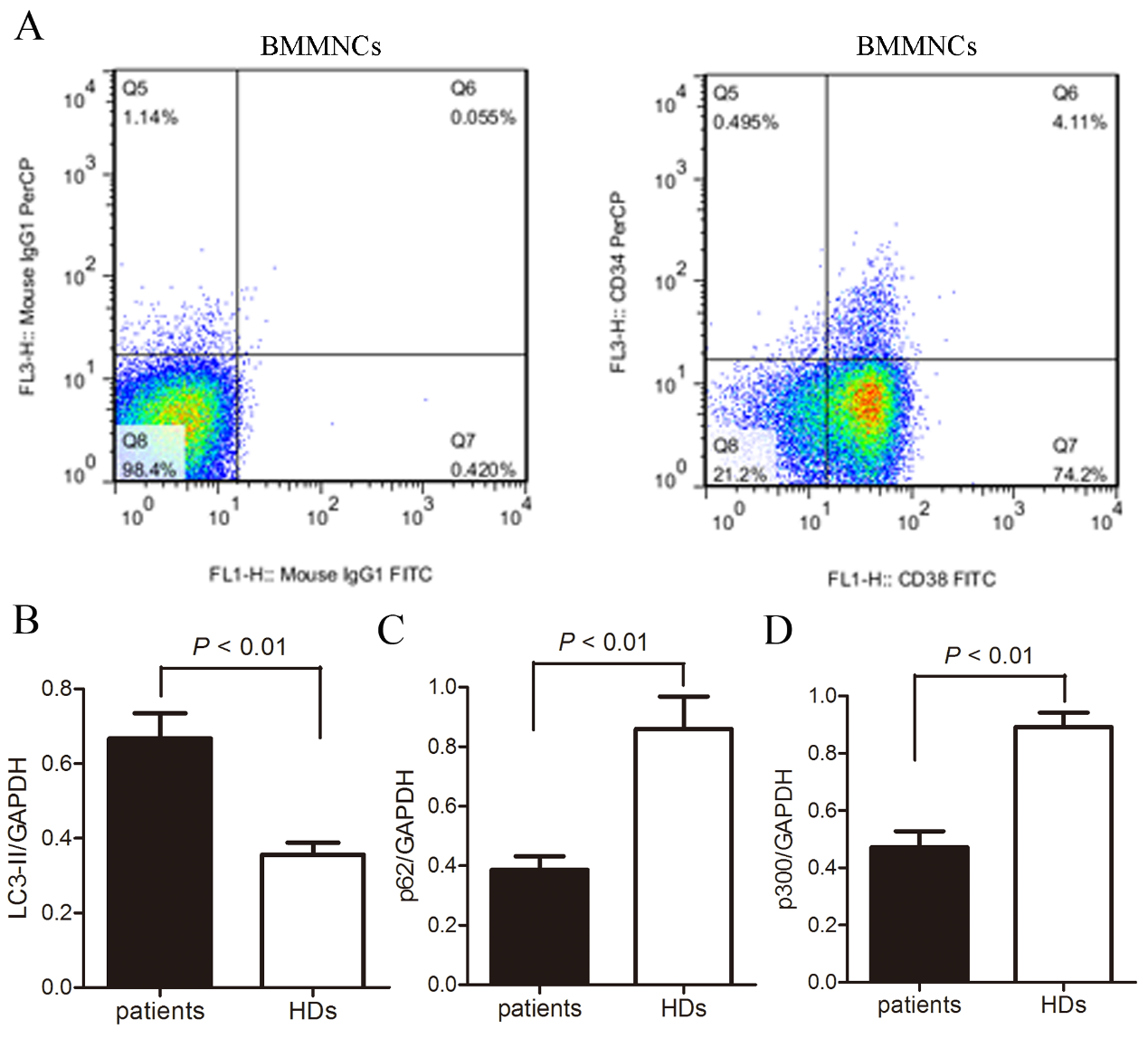
Figure S1.** Increased autophagy and decreased p300 expression in BMMNCs from patients with chronic benzene exposure. (**A**) BMMNCs mainly consisted of hematopoietic progenitor cells using flow cytometric analyses of CD34 and CD38 expression. (**B** - **D**) Statistical data showed that increased transition of LC3-II from LC3-I, decreased p62 and p300 expression in BMMNCs from patients with chronic benzene exposure (n=7) compared with those from healthy donors (HDs, n=7).

**
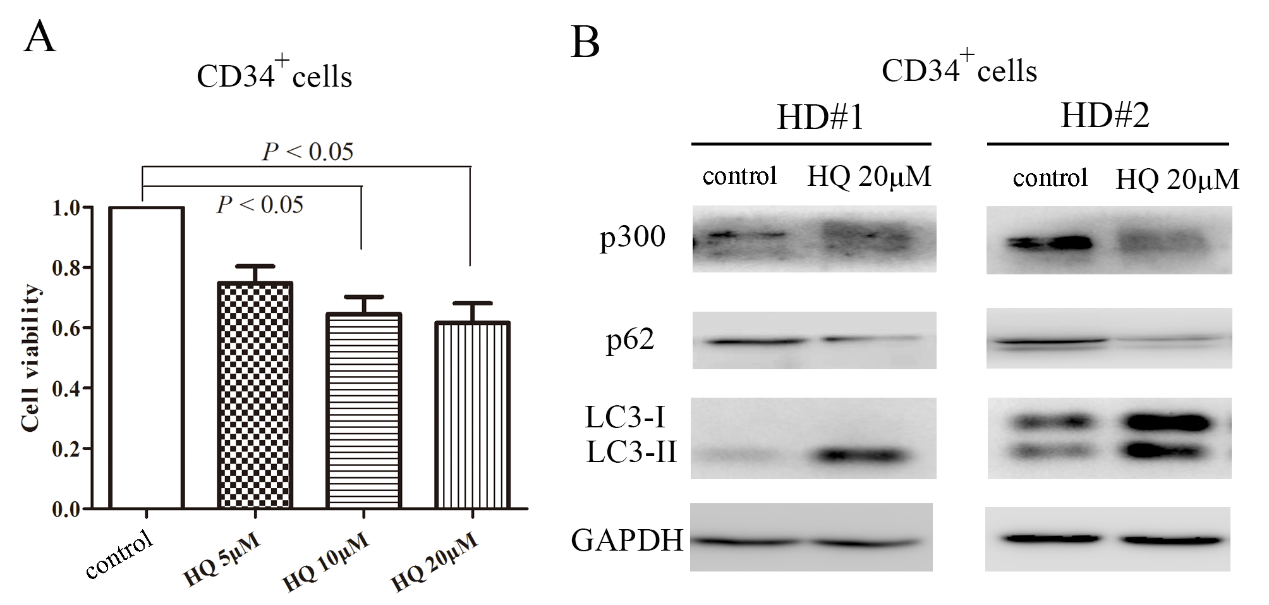
**

**Figure S2.** HQ treatment for 12 hours inhibits the cell viability and p300 expression, and induces autophagy in CD34^+^ hematopoietic stem and progenitor cells. (**A**) The cell viability was evaluated by trypan blue exclusion assay in CD34^+^ cells isolated from bone marrow of three healthy donors. (**B**) The expression of p300, p62 or LC3 was determined using western blot assay in CD34^+^ cells isolated from bone marrow of two healthy donors.


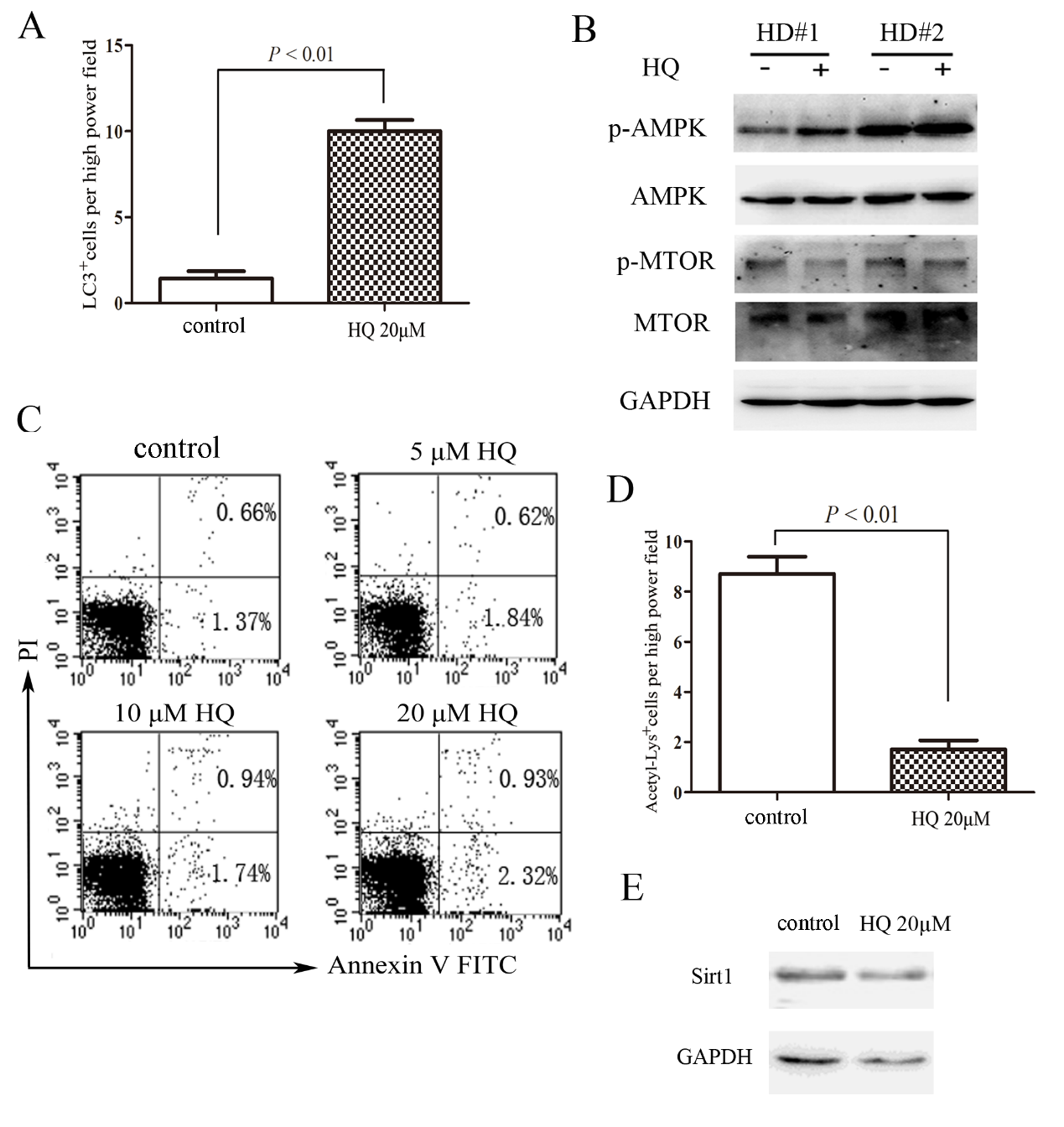


**Figure S3.** HQ increases autophagy flux with decreased acetylation level and almost does not influence apoptosis in BMMNCs. (**A**) Treatment with 20 μM HQ for 12 hours significantly enhanced the LC3 expression in BMMNCs using immunofluorescence. Statistical data were carried out in 20 randomly chosen high-power fields (× 400). (**B**) Treatment with 20 μM HQ for 12 hours promoted the phosphorylation of AMPK and inhibited the phosphorylation of MTOR in BMMNCs isolated from two healthy donors. (**C**) BMMNCs were treated with various concentrations of HQ for 12 hours, and stained with annexin V-FITC and PI, and then apoptotic cells were quantified on a flow cytometry. (**D**) Treatment with 20 μM HQ for 12 hours significantly reduced the acetylated lysine epitopes in BMMNCs using immunofluorescence with antibody against acetyl-lysine. Statistical data were carried out in 20 randomly chosen high-power fields (× 400). (**E**) Treatment with 20 μM HQ for 12 hours almost did not influence the expression of Sirt1 in BMMNCs.


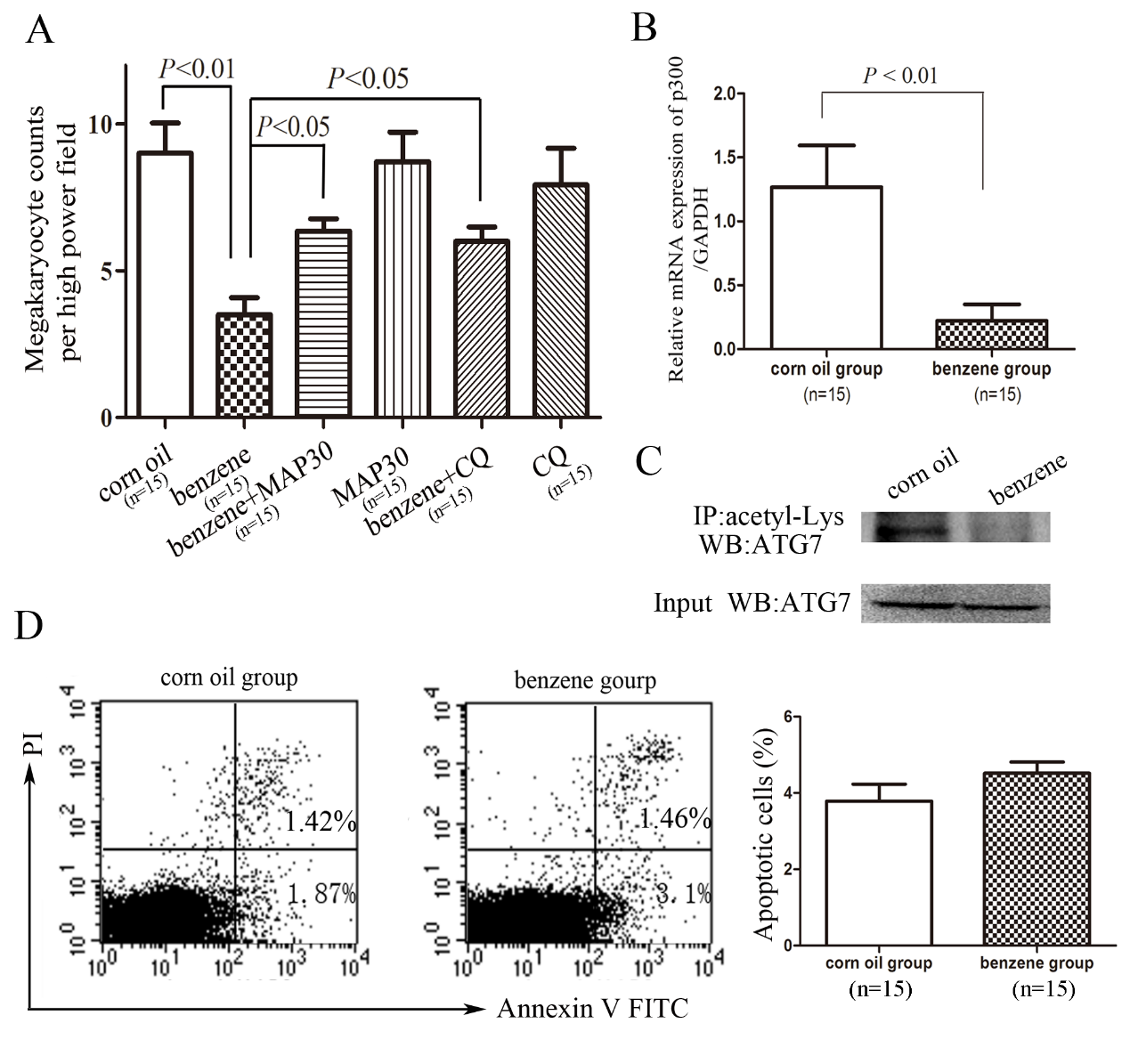


**Figure S4.** Benzene treatment results in reduced megakaryocytes, decreased p300 mRNA expression and acetylation of ATG7, and almost does not influence apoptosis in a benzene-induced hematotoxicity model. (**A**) The megakaryocytes were counted in the femurs from six group mice using HE staining. (**B**) Chronic exposure to benzene significantly inhibited the expression of p300 at the transcription level. Total RNA was extracted from BMMNCs and transcribed into cDNA and quantitative real-time PCR using specific primers of p300. (**C**) The endogenous acetylation of ATG7 was analyzed by IP with the antibody of acetyl-lysine, followed by western blot of ATG7 in BMMNCs from corn oil- or benzene-treated mice. Images representing four independent experiments were shown. (**D**) Bone marrow was flushed out using RPMI 1640 and mononuclear cells were separated using Ficoll. Then, apoptosis was analyzed using the annexin V/PI double staining method on a flow cytometry. Representative dot plots and statistical data of apoptosis cells were shown.
